# Identification and characterisation of a major outer membrane protein from *Methylacidiphilum fumariolicum* SolV

**DOI:** 10.1007/s10482-023-01879-0

**Published:** 2023-09-22

**Authors:** Changqing Liu, Rob Mesman, Arjan Pol, Federica Angius, Huub J. M. Op den Camp

**Affiliations:** Department of Microbiology, Radboud Institute for Biological and Environmental Sciences, Faculty of Science, Radboud University, Nijmegen, The Netherlands

**Keywords:** Methanotroph, Outer membrane protein, Methylacidiphilum, Cell wall

## Abstract

The outer membrane (OM) protects Gram-negative bacteria against a hostile environment. The proteins embedded in the OM fulfil a number of tasks that are crucial to the bacterial cell. In this study, we identified and characterised a major outer membrane protein (WP_009059494) from *Methylacidiphilum fumariolicum* SolV. PRED-TMBB and AlphaFold2 predicted this protein to form a porin with a β-barrel structure consisting of ten antiparallel β-sheets and with a small amphipathic N-terminal α-helix in the periplasm. We purified soluble recombinant protein WP_009059494 from *E. coli* using Tris–HCl buffer with SDS. Antibodies were raised against two peptides in the two large extracellular loops of protein WP_009059494 and immunogold localisation showed this protein to be mainly present in the OM of strain SolV. In addition, this protein is tightly associated with the OM, and is resistant to extraction. Only a small amount can be isolated from the cell envelope using harsh conditions (SDS and boiling). Despite this resistance to extraction, WP_009059494 most likely is an outer membrane protein. A regular lattice could not be detected by negative staining TEM of strain SolV and isolated protein WP_009059494. Considering the specific ecological niche of strain SolV living in a geothermal environment with low pH and high temperatures, this major protein WP_009059494 may act as barrier to resist the extreme conditions found in its natural environment. In addition, we found an absence of the BamB, BamC and BamE proteins of the canonical BAM complex, in *Methylacidiphilum* and *Methylacidimicrobium* species. This suggests that these bacteria use a simple BAM complex for folding and transport of OM proteins.

**Supplementary Information:**

The online version contains supplementary material available at 10.1007/s10482-023-01879-0.

## Introduction

The cell envelope of Gram-negative bacteria consists of an inner membrane (IM) and an outer membrane (OM), which are separated by periplasm. Within the periplasm a thin peptidoglycan layer is present. The composition of the two membranes is significantly different. The IM consists of a symmetrical phospholipid bilayer containing mainly α-helical proteins, whereas the outer membrane is asymmetrical and contains mainly ß-barrel folded proteins (Horne et al. 2020). In the OM, the phospholipids are located in the inner leaflet, while lipopolysaccharides (LPS) are located in the outer leaflet. Besides LPS, this OM contains integral membrane proteins called outer membrane proteins (OMPs). LPS from Gram-negative bacteria typically consists of three parts: the hydrophobic lipid A (endotoxin), a phosphorylated and non-repetitive core oligosaccharides (core-OS) and an O-antigen (O-polysaccharide) (Raetz and Whitfield 2002). The main function of LPS is not only to maintain cell integrity but also during infections will trigger the host's immune response.

Approximately 2–3% of the genes present in Gram-negative bacterial genomes encode OMPs, which are integral membrane proteins with a ß-barrel structure, short loops between the strands in the periplasm and large extended loops at the extracellular side (Wimley 2003). So far, the structures of almost all such proteins display an even number of antiparallel ß-sheets. This structural feature contributes to their high stability in the membrane and helps them to withstand the harsh and constantly changing environment (Koebnik 2002). Since OMPs are exposed on the outside of the bacterial cell and are the first line of contact between the bacteria and external environment, OMPs carry out diverse functions. This includes maintenance of cell structural integrity (*e.g.* OmpA) (Koebnik 2002; Reusch 2012), adhesion factors for virulence (*e.g.* OmpX, NspA) (Fox et al. 2014; Heffernan et al. 1994; Koebnik 2002; Martin et al. 2016; Sadarangani et al. 2011), channels for nutrient uptake (*e.g.* LamB and ScrY) (Koebnik 2002), general porins (*e.g.* OmpF, OmpC and PhoE) (Vergalli et al. 2020), TonB-dependent receptors (*e.g.* FhuA and FepA) (Koebnik 2002), and enzymes such as proteases and lipases (*e.g.* OMPLA, DegP and DegQ) (Fu et al. 2018; Kolmar et al. 1996; Wang et al. 2016).

Besides, some OMPs integrated in the OM are capable of forming a surface layer that completely covers the microorganism. However typical S-layers, one of the most common surface structures in almost all Archaea and in many types of Bacteria do not have a ß-barrel structure (Sleytr et al. 2014). S-layers are mostly composed of identical protein or glycoprotein species, with molecular masses ranging from 40 to 170 kDa (Claus et al. 2005; Messner et al. 2010; Sleytr et al. 2014). S-layers exhibit regular and highly porous arrays with oblique (p1, p2), square (p4) or hexagonal (p3, p6) lattice symmetry (Sára and Sleytr 2000; Sleytr et al. 2014). In addition, S-layers are considered to be one of the most abundant OMPs, since they make up approximately 10% of cellular proteins in Archaea and Bacteria (Sleytr et al. 2014). The abundance and localization of S-layer provide a protective coat for microorganisms against harsh environment (Gerbino et al. 2015; Sára and Sleytr 2000; Sleytr et al. 2014).

*Methylacidiphilum fumariolicum* SolV, a representative of the verrucomicrobial methanotrophs, was isolated from a mudpot in the Solfatara volcano near Naples (Italy) (Pol et al. 2007). *M. fumariolicum* SolV grows optimally at pH 2.7 and 55 °C, and can assimilate CO_2_ using the Calvin-Benson-Bassham cycle (CBB) and fix N_2_ gas (Khadem et al. 2010, 2011, 2012). Methane can be used as energy source, but strain SolV can also use hydrogen and hydrogen sulphide as energy sources (Mohammadi et al. 2017; Schmitz et al. 2020, 2023). Although we have a preliminary understanding of the physiological properties of strain SolV, the proteins involved in its cell structure remain unknown. Upon analysis of the genome and transcriptomic data of *M. fumariolicum* SolV from different growth conditions, two highly expressed genes were attracting attention. One is the gene encoding a lipoprotein (protein ID: WP_009060351) characterised before (Liu et al. 2023). The other gene *MFUM_2087* is encoding an outer membrane protein WP_009059494, which ranks first among all outer membrane proteins and is characterised in this study.

## Materials and method

### Bioinformatic analysis

To find homologs of the OMP WP_009059494 in other bacteria, the amino acid sequence of WP_009059494 was used in a BlastP search. The signal peptide of WP_009059494 was predicted by SignalP 6.0 using the standard settings for Gram-negative bacteria (Teufel et al. 2022). The molecular mass of WP_009059494 (without predicted signal peptide) was calculated using the ‘compute pI/Mw’ tool in ExPASy (Gasteiger et al. 2005). For the secondary structure prediction of WP_009059494, PSIPRED 4.0 was used (Buchan et al. 2013). The structure of WP_009059494 was predicted by using PRED TMBB (Bagos et al. 2004) and AlphaFold 2 (Jumper et al. 2021). Subcellular localization was determined using PSORTb v3.0 (set for Gram-negative bacteria) (Yu et al. 2010).

### Expression of protein WP_009059494 in *E. coli*

The gene *MFUM_2087* was optimized for the preferred codon usage of *E. coli* and synthesized by Biolegio Company (Nijmegen, The Netherlands) with pET-28a as the vector (pET-28a-*MFUM_2087*). Since the protein encoded by *MFUM_2087* was predicted to be an OMP by bioinformatic analysis, a second construct with the PelB signal peptide from *E. coli* replacing the original signal peptide of *MFUM_2087* was produced (pET-28a-PelB-*MFUM_2087*).

Three plasmids, pET-28a, pET-28a-*MFUM_2087*, and pET-28a-PelB-*MFUM_2087* were transformed into the expression strain *E. coli* Rosetta™ 2(DE3) and transformants were precultured overnight in 5 mL of LB medium with appropriate antibiotics at 37 °C and 250 rpm. Then, 1 ml of the seed culture was transferred into 1 l Erlenmeyer flasks containing 600 mL of LB medium, and flasks were incubated at 37 °C and 200 rpm. When the cell density reached OD_600_ of 0.6–0.8, the cultures were induced with isopropyl-β-D-thiogalactopyranoside (IPTG) to a final concentration of 1 μM. After a further overnight incubation at 28 °C, cells were collected.

### Purification of protein WP_009059494 from *E. coli*

Cells were harvested and washed with phosphate-buffered saline (PBS 10 mM, pH 7.4) and then centrifuged at 5,000 g for 20 min (4 °C). The cell pellets were resuspended in Buffer A ((50 mM Tris–HCl pH 8.0, 5 mM MgCl_2_, 1 mM dithiothreitol) with EDTA-free protease inhibitor cocktail (cOmplete EDTA-free, Roche) and disrupted by two passes through a French pressure cell (120 MPa). The lysed cell suspension was clarified by centrifugation for 30 min at 10,000 g, 4 °C. The supernatant was analysed by SDS-PAGE. Unbroken cells, large cellular debris, and inclusion bodies were pelleted. The pellets were dissolved using Tris–HCl buffer containing 4 M urea, 4 M guanidine HCl or 0.4% SDS and lysed using a tissue homogenizer. The suspensions were then centrifuged for 30 min at 10,000 g, 4 °C. This supernatant was also analysed by SDS-PAGE.

20 mM SDS was found to be more effective in dissolving the inclusion bodies than urea and guanidine HCl. Therefore inclusion bodies were dissolved as follows: first, inclusion bodies were washed twice times with Buffer B (20 mM Tris–HCl, pH 7.4, 500 mM NaCl, 4 M urea) using a tissue homogenizer. The suspension was centrifuged for 30 min at 10,000 g, 4 °C. The supernatant was discarded and the pellet was resuspended in Buffer C (20 mM Tris–HCl, pH7.4, 500 mM NaCl, 0.1% ß-mercaptoethanol, 20 mM SDS). After this, the denatured protein was refolded using a dialysis bag with cut-off of 12 kDa with Buffer D (20 mM Tris–HCl, pH7.4, 500 mM NaCl, 0.1% ß-mercaptoethanol, 0.1% 2-hydroxyethyl disulphide, 0.1% SDS) overnight, followed by Buffer E (20 mM Tris–HCl, pH7.4, 500 mM NaCl, 0.1% ß-mercaptoethanol, 0.1% 2-hydroxyethyl disulphide, 5 mM ß-cyclodextrin) for 6 h; and finally, a 30 kDa cut-off spin filter (Vivaspin; Sartorius Stedim Biotech) was used to remove ß-cyclodextrin by dilution and concentration with 20 mM Tris–HCl (pH 7.4).

### Purification of protein WP_009059494 from strain SolV biomass

*M. fumariolicum* SolV was cultured in a continuous bioreactor under methane limitation as described previously (Schmitz et al. 2023). Cells were harvested, washed twice with phosphate-buffered saline (PBS) and resuspended in Buffer F (10 mM sodium phosphate, pH 7.4 and 100 mM NaCl). The cells were lysed by three passes through a French pressure cell (120 MPa). The protein WP_009059494 was extracted from strain SolV following the scheme depicted in the Results section based upon a protocol for *E. coli*. The supernatant and pellet obtained at each step were collected and analysed by SDS-PAGE and Western blot. The lysed cell suspension was centrifuged at 8000 g for 30 min at 4 °C to obtain supernatant and pellet. Total membrane was collected by centrifugation of the supernatant at 30,000 g for 1 h at 4 °C and resuspended in Buffer G (10 mM sodium phosphate, pH 7.4, 100 mM NaCl, and 2% N-lauroylsarcosine (Sarkosyl, Sigma) to dissolve the IM. Supernatant containing IM proteins was obtained by ultracentrifugation at 30,000 g for 1 h at 4 °C. The pellet was washed twice and was resuspended in Buffer H (10 mM sodium phosphate pH 7.4, 200 mM NaCl, 50 mM EDTA,10 mM DTT and 20 μg lysozyme ml^−1^ and 2% CHAPS) and incubated at room temperature (RT) overnight. The supernatant containing OM proteins was collected after ultracentrifugation at 30,000 g for 1 h, 4 °C, then diluted to 0.5% CHAPS, and concentrated using 30-kDa cutoff spin filters (Vivaspin; Sartorius Stedim Biotech). The insoluble materials were resuspended in a phosphatebuffer with SDS (10 mM sodium phosphate, pH 7.4, 300 mM NaCl and 0.4% SDS) and boiled for 30 min. Boiling with SDS was used to remove cellular components that were not covalently bound to the peptidoglycan. After incubation at RT overnight, the supernatant containing SDS soluble protein was collected after centrifugation at 30,000 g for 1 h, 4 °C. Pellets containing cell debris and unbroken cells were treated following the same protocol. In addition, a protocol described before (van Teeseling et al. 2014a) for S-layer enrichment was applied. Briefly, concentrated cells were resuspended in 20 mM HEPES buffer (pH 7.5) (including 15 mM NaHCO3, 2 mM CaCl2, and 0.8 mM MgSO4), after which the protease inhibitor phenylmethylsulfonyl fluoride (PMSF) and DNase II were added. The cells were left at room temperature (RT) for 20 min and after this, the detergent Triton X-100 was added to a final concentration of 0.5% (vol/vol). After an incubation for 30 min at RT, the enriched surface proteins were pelleted and resuspended in the HEPES buffer.

### SDS-PAGE and MALDI-TOF MS

SDS-PAGE was performed using a 14% (w/v) resolving gel and a 5% (w/v) stacking gel. Proteins were visualized by staining with Coomassie Brilliant Blue-R250. Bands cut out from the SDS-PAGE gels were prepared for MALDI-TOF MS analysis. The samples were digested with trypsin as previously described (van Teeseling et al. 2014a). Afterwards, the samples were applied to a MALDI plate using α-cyano-hydroxy cinnamic acid as matrix and analysed on a Bruker Microflex® MALDI-TOF MS instrument. Proteins were identified using the MASCOT search tool (Matrix Science, London, United Kingdom) and a *M. fumariolicum* SolV protein database (https://mage.genoscope.cns.fr/microscope/home/index.php).

### Antibody generation and immunoblotting

The recombinant protein PelB-MFUM_2087 band was cut out from the SDS-PAGE gel and sent to Eurogentec (Belgium) for immunization of rabbits. In addition, two peptides selected from the WP_009059494 protein sequence were synthesized and used to generate antibodies. Antibodies were generated by immunization of a rabbit in a 3 months program. Pre-immunization sera used as control were collected before initiating the immunization.

Proteins on SDS-PAGE gels were transferred to nitrocellulose membranes using semi-dry blotting as described previously (van Teeseling et al. 2014a). Blots were incubated in ddH_2_O for 30 min, and afterwards for 60 min in 2% skimmed milk powder (Frema Reform, Lüneburg, Germany) in Tris-buffered saline (TBS, 10 mM Tris–HCl pH 7.4, including 137 mM NaCl and 2.7 mM KCl). Blots were then incubated for 60 min with the antisera and pre-immune serum (control) diluted 1000-fold in blocking buffer. After incubation, blots were subsequently washed three times for 10 min in TBS containing 0.05% Tween 20. After this step, blots were incubated with a secondary antibody (anti-rabbit IgG alkaline phosphatase (Sigma, Zwijndrecht, The Netherlands), diluted 30,000 times) for 60 min, followed by washing twice for 10 min with TBS containing 0.05% Tween 20 and twice for 10 min with TBS. Finally, blots were incubated with 5-bromo-4-chloro-3-indole phosphate (BCIP)/nitroblue tetrazolium (NBT) liquid substrate system (Sigma, Zwijndrecht, The Netherlands) for 150 s and rinsed with ddH_2_O for 10 min. All steps were performed at room temperature and all lanes were imaged with the same settings.

### Immunogold localization

Immunogold localization was performed as previously described (van Teeseling et al. 2018). For immunogold labelling on grids with ultrathin sections of strain SolV cells (High-pressure frozen, freeze substituted in anhydrous acetone with 0.2% UA followed by low temperature (−50 °C) embedding with lowicryl HM20 and polymerization at low temperature (−50 °C) using long wavelength UV irradiation), the grids were first washed for 2 min on 5 drops of 0.1 M PHEM buffer (60 mM piperazine-N, N′-bis (2-ethanesulfonic acid) (PIPES), 25 mM HEPES, 10 mM EGTA, 2 mM MgCl_2_ (pH 6.9)) and then blocked by incubation for 15 min on 0.1 M PHEM containing 1% BSA. The incubation with the primary antibody was performed for 60 min with 200-fold diluted antiserum or preimmune serum (control) in 0.1 M PHEM containing 1% BSA. Washing was performed for 2 min on 5 subsequent drops of 0.1 M PHEM containing 0.1% BSA. The grids were then incubated for 45 min on protein A-coupled 10 nm gold particles (PAG-10; CMC UMC Utrecht) diluted 60-fold in 0.1 M PHEM containing 1% BSA. Washing for 45 s was performed on five drops of 0.1 M PHEM containing 0.1% BSA, followed by washing on five drops of 0.1 M PHEM for 2 min. Fixation was achieved by incubation on 1% glutaraldehyde in 0.1 M PHEM for 5 min, followed by washing the grids on 10 drops of MilliQ water (MQ) (1 min incubation per drop). Sections were stained on 2% uranyl acetate in MQ for 5 min, after which the grids were washed on 5 drops of MQ (1 min incubation per drop) and air dried. Grids containing ultrathin sections were examined in a JEOL JEM-1400 flash (Tokyo, Japan) transmission electron microscope (TEM) at 120 kV. Images were captured using the Matataki Flash sCMOS detector.

### Negative stain electron microscopy

The sample (from Lane 2, Fig. 4B) was attained as described above and adsorbed onto formvar/carbon-coated copper grids. The sample was negatively stained with 1% uranyl acetate, and visualized in a JEOL JEM-1400 flash (Tokyo, Japan) transmission electron microscope (TEM) at 120 kV.

### NPN uptake assay, diSC3(5) release assay and spot analysis

DiSC_3_(5) (dipropylthiadicarbocyanine iodide) release assays and NPN (1-N-phenylnapthylamine) uptake assays were performed according to Konovalova et al. (2016) (Konovalova et al. 2016). Overnight cultures of *E. coli* Rosetta™ 2(DE3) containing different plasmids were serial diluted and spotted on LB agar plates containing kanamycin and chloramphenicol, supplemented with or without 0.01/0.02% SDS and 1 mM EDTA, and incubated overnight at 37 °C.

## Results

### Transcriptome and bioinformatic analysis

Transcriptomic data previously obtained from *M. fumariolicum* SolV cells cultivated under different growth conditions (CH_4_/NH_4_^+^, CH_4_/NO_3_^−^ and H_2_/NH_4_^+^) showed that several genes were highly expressed, including key genes involved in methane oxidation (*e.g*. *pmoABC* and *xoxF*) and the carbon-fixing CBB cycle (*e.g*. *cbbL* and *cbbS*) (Mohammadi et al. 2017). Our attention was drawn to the high expression of genes encoding two unknown proteins. One of them is WP_009060351 (gene: *MFUM_0397*), which ranks first among all lipoproteins and has been characterised recently (Liu et al. 2023). This lipoprotein might play a vital role in the linkage between the OM and the peptidoglycan. The other protein is WP_009059494 (gene: *MFUM_2087*), a putative outer membrane protein, which gene expression ranks first among all outer membrane proteins (Supplementary Table S1) and is characterised in this study.

**Fig. 1:**
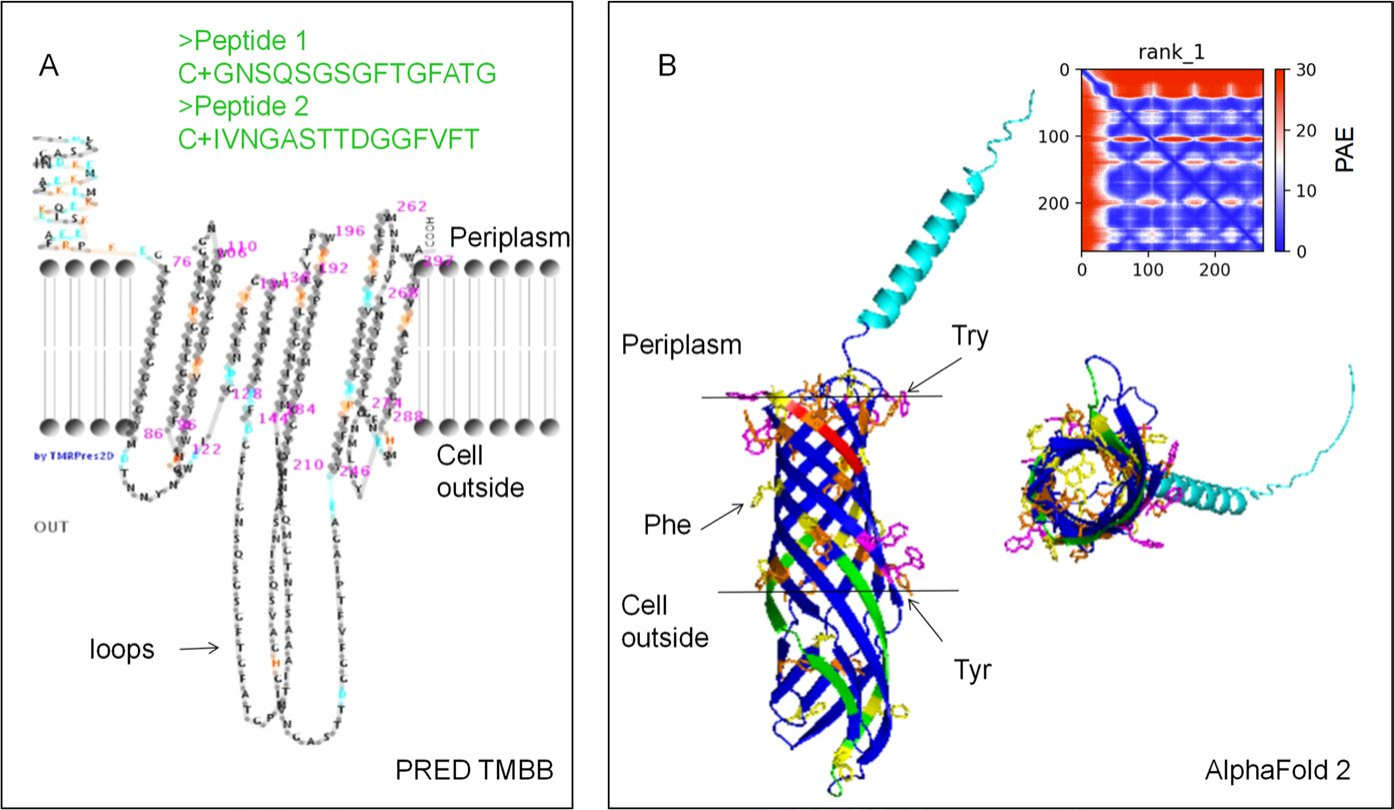
Structure prediction of protein WP_009059494 (*MFUM_2087*) from strain SolV. The signal peptide at the N-terminus of WP_009059494 is not present in the predicted structure. **A** A Hidden Markov Model method predicted WP_009059494 as ß-barrel OMP using PRED TMBB. Peptide 1 and peptide 2 from the loops were used to generate antibodies. **B** Model generated by AlphaFold 2. The N-terminus forming a short α-helix is indicated in cyan and the C-terminus is shown in in red. Peptide 1 and peptide 2 used for generating antibodies are indicated in Green. The picture at the right shows a view into the protein from the extracellular space. Aromatic amino acids (Try, Tyr and Phe) were marked. The inter PAE (Predicted Aligned Error) between chains is very low indicating a confident prediction

**Fig. 2:**
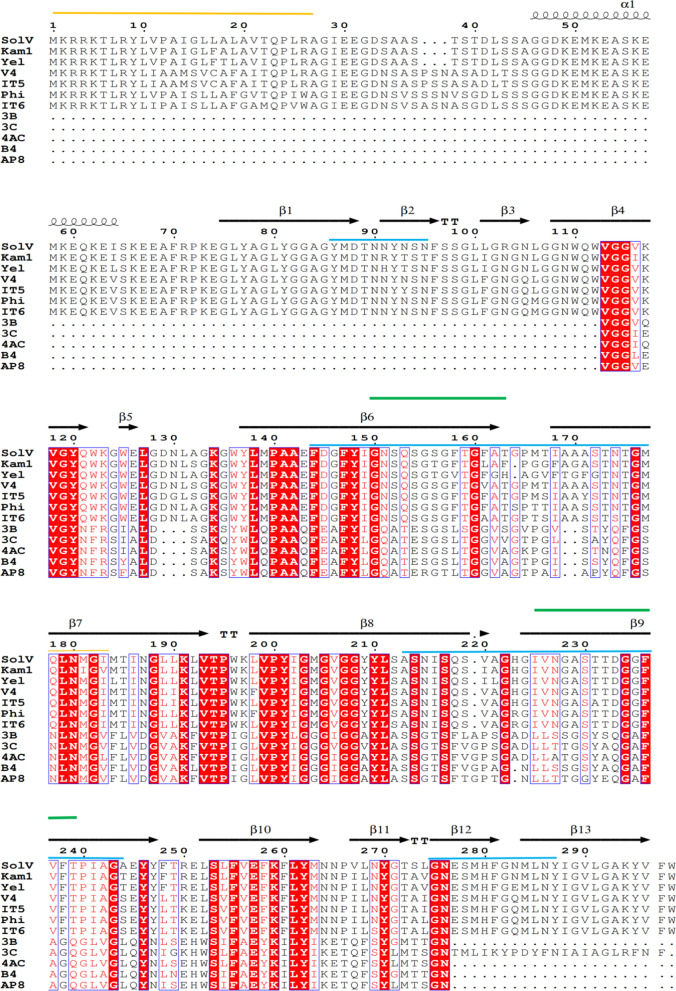
Protein sequence analysis and alignment of WP_009059494 (*MFUM_2087)* with its orthologs. Homology analysis of amino acid sequences in *Methylacidiphilum* and *Methylacidimicrobium* species. From top to bottom: Strain SolV, *Methylacidiphilum kamchatkense* (WP_039720966.1), *Methylacidiphilum* sp. Yel (WP_134389778.1), *Methylacidiphilum infernorum* V4 (WP_012462709.1), *Methylacidiphilum sp*. IT5 (WP_206845119.1), *Methylacidiphilum* sp. Phi (WP_134439559.1), *Methylacidiphilum* sp. IT6 (WP_206825941.1), *Methylacidimicrobium cyclopophantes* 3B (WP_142524243.1), *Methylacidimicrobium fagopyrum* 3C (WP_020494287.1), *Methylacidimicrobium tartarophylax* 4AC (WP_142659387.1), *Methylacidimicrobium sp*. B4 (WP_206863730.1) and *Methylacidimicrobium thermophilum* AP8 (WP_202213476.1). Secondary structure was marked by the prediction of AlphaFold 2 (arrows), and aligned amino acid residues conserved in all proteins are highlighted in red. Signal peptide regions, extracellular loops and synthetic peptides are marked by yellow, blue and green lines, respectively

WP_009059494 contains 298 amino acids, of which the 26 residues at N-terminus were predicted to be a Sec signal peptide with high confidence (93.7%) by the SignalP 6.0 server using the standard settings for Gram-negative bacteria (Teufel et al. 2022). The predicted molecular masses are about 32 kDa with and 29 kDa without signal peptide, and the theoretical isoelectric point is 6.75. Amino acid analyses revealed this protein contains 41% hydrophobic amino acids, 7.7% acidic amino acids and 8.4% basic amino acids. The gene encoding WP_009059494 is a separate gene and not part of an operon. The PRED-TMBB server predicted that the WP_009059494 without its signal peptide forms a β-barrel OMP and suggested 10 transmembrane domains with two large extracellular loops toward the outside of the cell and an N-terminal flexible region in the periplasm (Fig. 1A). Two short amino acid sequences in the two large extracellular loops were synthesized and used to generate antibodies for further experiments. The three-dimensional structure was predicted using AlphaFold2 (Fig. 1B). This model showed WP_009059494 as an OMP with ten-β-sheets and an N-terminal α-helix flexible region (amino acids 46–64) in the periplasm. Furthermore, two large extracellular loops in this model form β-sheets toward the outside of the cell. A BlastP search gave no significant hits for WP_009059494 to characterised proteins, but showed that this protein is highly conserved in *Methylacidiphilum* species (Fig. 2). Interestingly, the N-terminal flexible region is only highly conserved in *Methylacidiphilum* species, while the remaining part of the protein is also conserved in *Methylacidimicrobium* species.

In *E. coli*, the BAM complex responsible to fold and insert the nascent OMPs into the OM, consists of BamA, the OMP itself, and four lipoproteins called BamB, BamC, BamD and BamE, all of which are anchored to the inner leaflet of the OM via an N-terminal post-translational lipid modification (Hagan et al. 2011; Ricci and Silhavy 2012). BamB and BamD interact directly with BamA, and BamC and BamE interact directly with BamD (Kim et al. 2007). BamA and BamD form the core of the complex and are the essential components required for cell viability (Wu et al. 2005). Although the composition of the BAM complex varies from bacterial species to bacterial species, for example, *N. meningitidis* appears to lack BamB (Volokhina et al. 2009). BlastP searches revealed the presence of BamA (*MFUM_1849*) and BamD (*MFUM_1939*) encoding genes in strain SolV, while genes encoding BamB, BamC and BamE were absent also in other *Methylacidiphilum* and *Methylacidimicrobium* species genomes (Table 1) This indicates that they may use a simple BAM complex system. In addition, lipoprotein BamD and LptE are located in an operon without other proteins. LptE is one component of the LPS export pathway lptABCDEFG, transporting LPS from the IM to the cell surface. These seven proteins are essential for the cell viability. When any one of the Lpt proteins is depleted, de novo synthesized LPS can no longer reach the cell surface and abnormal membrane structures accumulate in the periplasm (Ruiz et al. 2008; Sperandeo et al. 2007, 2008). BlastP searches revealed all of them are present in strain SolV and other *Methylacidiphilum* and *Methylacidimicrobium* species, indicating their importance and conservation..

**Table 1:** BlastP analysis to identify proteins involved in β-barrel assembly and LPS transport in verrucomicrobial methanotrophs

	*E. coli*	SolV	Kam1	Yel	V4	Phi	3B	AP8	4AC
β-barrel assembly	BamA	Mfum_1849 25%	kam1_1557 99%	LXQB01_90011 98%	Minf_0751 97%	LXQC01_1400018 95%	CABFUZ02_80002 77%	MTHMO_1104 70%	CABFVA02_1140050 74%
	BamB	–	–	–	–	–	–	–	–
	BamC	–	–	–	–	–	–	–	–
	BamD	Mfum_1939	kam1_1620 99%	LXQB01_10012 99%	Minf_1577 87%	LXQC01_1760033 92%	CABFUZ02_2560023 68%	MTHMO_0970 67%	CABFVA02_420002 66%
	BamE	–	–	–	–	–	–	–	–
LPS transport	LptA	Mfum_1152 27%	kam1_982 99%	LXQB01_70015 98%	Minf_0952 85%	LXQC01_1540023 91%	CABFUZ02_2610006 62%	MTHMO_1083 63%	CABFVA02_1140030 60%
	LptB	Mfum_1106 50%	kam1_944 98%	LXQB01_110053 95%	Minf_0911 94%	LXQC01_1540065 89%	CABFUZ02_790015 66%	MTHMO_0944 66%	CABFVA02_110015 75%
	LptC	Mfum_1153	kam1_983 98%	LXQB01_70016 95	Minf_0953 78%	LXQC01_1540022 80%	CABFUZ02_2610005 57%	MTHMO_1084 55%	CABFVA02_1140031 59%
	LptE	Mfum_1938	kam1_1619 97%	LXQB01_10013 99%	Minf_1576 86%	LXQC01_1760032 99%	CABFUZ02_2560022 62%	MTHMO_0971 62%	CABFVA02_420003 62%
	LptF	Mfum_2323 19%	kam1_2001 99%	LXQB01_330036 100%	Minf_2191 93%	LXQC01_1360071 98%	CABFUZ02_1290013 71%	MTHMO_0109 71%	CABFVA02_20040 70%
	LptG	Mfum_0853 23%	kam1_709 99%	LXQB01_270047 98%	Minf_1642 85%	LXQC01_1350003 93%	CABFUZ02_950002 62%	MTHMO_0539 60%	CABFVA02_1260003 59%
	LptD	Mfum_0026	kam1_31 98%	LXQB01_670004 98%	Minf_0031 90%	LXQC01_1240087 94%	CABFUZ02_1350015 55%	MTHMO_0044 56%	CABFVA02_120084 57%

The amino acid sequences of BamA, B, C, D, E and LptA, B, F, G from *E. coli* K–12 substr. MG1655 were retrieved from GenBank and used for BLASTp searches against *Ca. Methylacidiphilum fumariolicum* SolV (taxid:591,154) genome, then the amino acid sequences of BamA, BamD and LptA, B,F, G from stain SolV were used for a second round of BLASTp searches against the whole genomes of verrucomicrobial methanotroph strains including *Methylacidimicrobium* (taxid:1,541,670), *Methylacidimicrobium tartarophylax* 4AC (taxid:1,041,768), *Methylacidimicrobium fagopyrum* 3C (taxid:1,134,055), *Methylacidimicrobium thermophilum* AP8 (taxid:2,730,359), *Methylacidimicrobium cyclopophantes* 3b (taxid:1,041,766), *Methylacidiphilum infernorum* V4 (taxid:481,448), *Methylacidiphilum kamchatkense* (taxid:431,057), *Methylacidiphilum sp.* Phi (taxid:1,847,729), *Methylacidiphilum sp*. Yel (taxid:1,847,730) (https://mage.genoscope.cns.fr/microscope/search/blast)

Using BamB, BamC, BamE, LptC, LptD and LptE from *E. coli* as query, not hits were obtained against these strains described above. For the BamD, BamD from *E. coli* was used for BLASTp searches against the Verrucomicrobia (taxid: 74,201), the hit LptC from *Chthoniobacterales bacterium* (MBA2431946.1, 25% identity to *E. coli*) was used for a second BLASTp search against the eight strains mentioned above. For LptE and LptE, protein sequences from Proterobacteria were used for a BLASTP search against the Verrucomicrobia (taxid: 74,201), the hit LptE from *Prosthecobacter dejongeii* (WP_184206993.1, 24% identity) was used for a second BLASTp search against the eight strains mentioned above. For LptC and LptC protein sequences from Proteobacteria were used for a BLASTp search against the Verrucomicrobia (taxid: 74,201), the hit LptC from *Chthoniobacterales bacterium* (MBE2204699.1, 33% identity) was used for a second BLASTp search against the eight strains mentioned above. For LptD and LptD protein sequences from *E. coli* were used for a BLASTp search against the Verrucomicrobia (taxid: 74,201), the hit LptD from a Verrucomicrobiota bacterium (MBV9488245.1, 27%) was used for a second BLASTp search. The hit for strain AP8 (23%) was used for a third round of BLASTp searches against the other strains

### Protein purification and immunoblotting results for *E. coli* and strain SolV

**Fig. 3:**
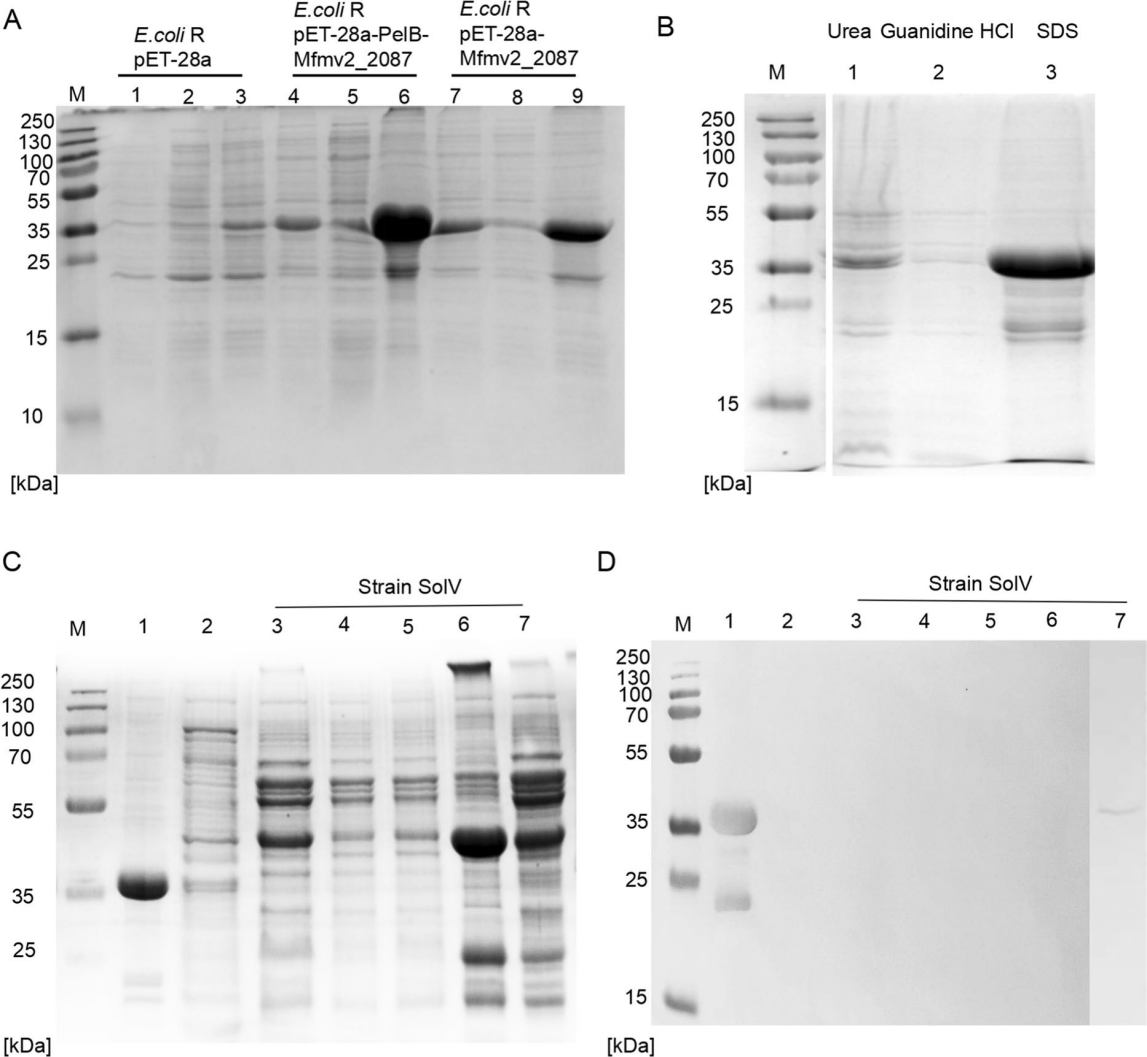
Overexpression of protein WP_009059494 (gene: *MFUM_2087*) in *E. coli* and immunoblot analysis. **A** SDS-PAGE of protein WP_009059494 in *E. coli*. Lane 1, 4, 7, represent total crude protein of *E. coli* containing empty plasmid pET-28a, plasmid pET-28a-PelB*-MFUM_2087* and plasmid pET-28a-*MFUM_2087*, respectively. Lane 2, 5, 8, represent protein in supernatant after centrifugation (10,000 g for 30 min). Lane 3, 6, 9, represent protein in the pellet after centrifugation (10,000 g for 30 min). **B** Proteins in the pellet (Lane 6), were solubilized with 4 M urea (Lane 1), 4 M guanidine HCl (Lane 2), and 0.4% SDS (Lane 3), respectively. **C** SDS-PAGE of protein in *E. coli* and different cell fractions from strain SolV. Lane 1, represents purified recombinant protein PelB-MFUM_2078; Lane 2, represents total crude extract from *E. coli* containing the empty plasmid pET-28a; Lane 3, represents total crude extract from strain SolV; Lane 4, represents protein in the supernatant from strain SolV after centrifugation of crude extract (10,000 g for 30 min); Lane 5, represents protein in the pellet from strain SolV after centrifugation of crude extract (10,000 g for 30 min); Lane 6, represents total membrane proteins from strain SolV; Lane 7, represents the sample obtained following the S-layer enrichment protocol described by van Teeseling et al. (2014a). **D** Immunoblot analysis using protein fractions from **C** showed that the antiserum (anti-PelB-MFUM_2078) reacts with recombinant protein PelB-MFUM_2078 (two bands around 35 kDa and 24 kDa, Lane 1) and with putative surface protein from strain SolV (Lane 7)

To construct the expression system in *E. coli* strain, the gene *MFUM_2087* with signal peptide was synthesized after adapting it to the preferred codon usage of *E. coli*. Then, expression vector pET-28a carrying the T7 promoter and lac-operator system was used to construct pET-28a-*MFUM_2087* (including the original peptide signal, 31 kDa) for the production of a recombinant protein. In a second construct, the PelB leader sequence, containing 22 amino acids, was used to replace the original signal peptide, resulting in pET-28a-PelB-*MFUM_2087* (32 kDa). This PelB leader sequence has been used in pET vector expression systems to obtain bioactive heterologous proteins. For example, the PelB signal was used to induce secretion of extracellular PETase in *E. coli* BL21 (Shi et al. 2021). Both constructs were used to express the encoded proteins in *E. coli*. In both cases, the expressed proteins were present in inclusion bodies (Fig. 3A, Lane 6 and 9). The SDS-PAGE showed a main band at around 35 kDa, which was slightly larger than the expected molecular mass (31–32 kDa). The detergents urea (4 M), guanidine HCl (4 M), and SDS (0.4%) were used to dissolve the inclusion bodies, and the results showed that only SDS could extract the protein from inclusion bodies (Fig. 3B). Using an inclusion bodies purification protocol, and refolding dialysis, purified PelB-MFUM_2087 was retrieved and analysed by SDS-PAGE (Fig. 3C, Lane 1). MALDI-TOF MS verified the band at around 35 kDa as WP_009059494. Surprisingly, the faint band around 20 kDa was also identified as the same protein by MALDI-TOF MS and most likely represents a partially degraded form.

To identify WP_009059494 in different fractions from strain SolV, antibodies were generated against purified recombinant protein PelB-MFUM_2087. The specificity of the antisera was verified by an immunoblot using purified PelB-MFUM_2087 showing that the antibody indeed bound PelB-MFUM_2087, as the seen by the signal at the expected molecular mass around 35 kDa (Fig. 3CD, Lane 1), and no signal was observed in the crude extract from the *E. coli* containing the empty plasmid (Fig. 3C, Lane 2). For the strain SolV fractions, no signal was present in total crude extract (Lane 3), supernatant (Lane 4), pellet (Lane 5) and total membrane fraction (Lane 6). A low signal intensity was found in the surface protein factions (Lane 7). The localization of WP_009059494 in strain SolV using anti-PelB-MFUM_2087 antibody immunogold transmission electron microscopy (TEM) did unfortunately not work.

**Fig. 4:**
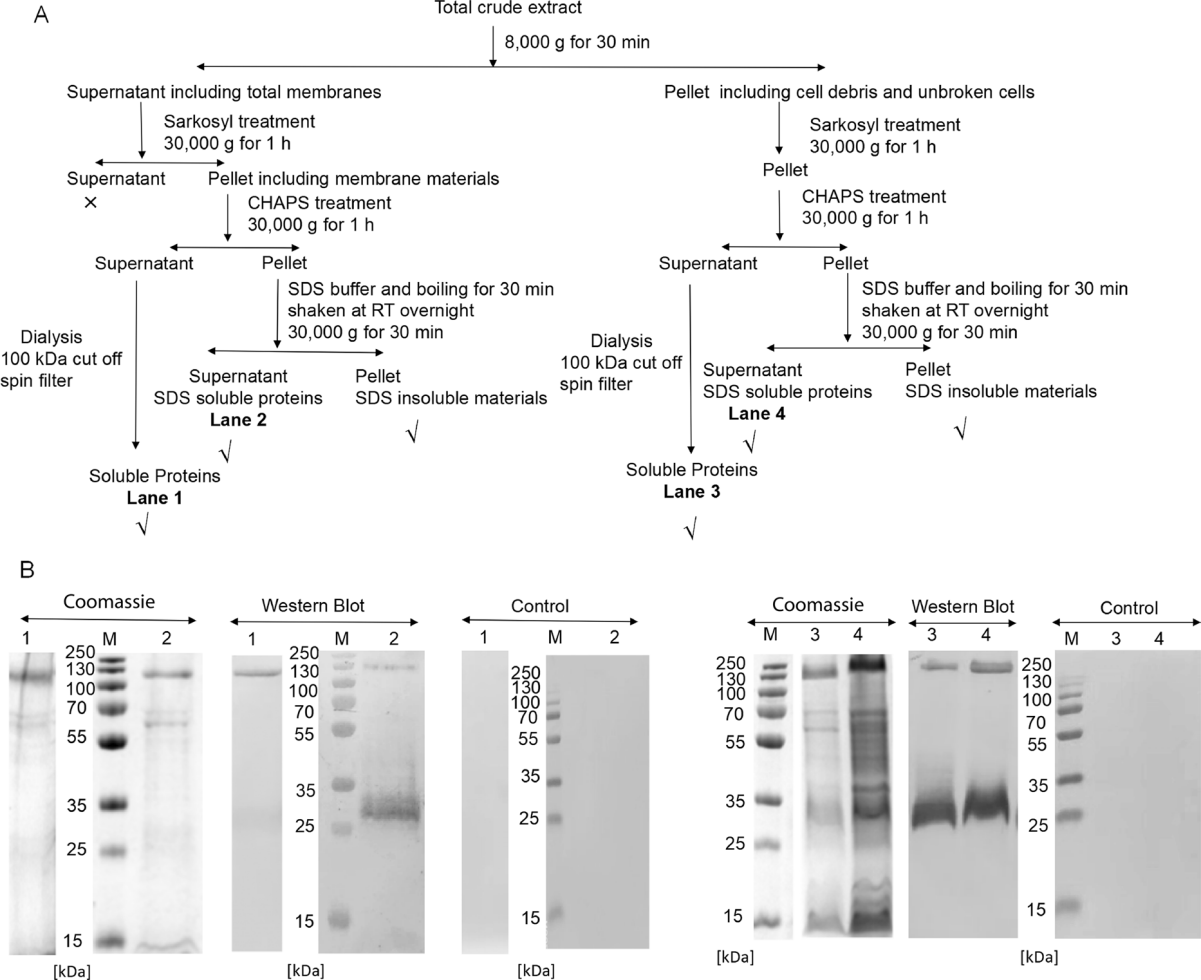
Purification of protein WP_009059494 (MFUM_2087) in strain SolV and immunoblot analysis. **A** Flow diagram for purified protein from strain SolV. √, represent protein fractions showing a reaction with antiserum raised against synthetic peptides; × , represent protein fraction that did not react with this antiserum. **B** Immunoblot analysis using soluble proteins from **A** all react with antiserum (anti-peptide 1/2). Lane 1, represents soluble protein after Sarkosyl and CHAPS treatment from total membranes. Lane 2, represents soluble protein after Sarkosyl, CHAPS and SDS treatment from total membranes. Lane 3, soluble protein after Sarkosyl and CHAPS treatment from cells debris and unbroken cells. Lane 4, represents soluble protein after Sarkosyl, CHAPS and SDS treatment from cells debris and unbroken cells. Controls were performed with pre-immune serum

Next, two short synthetic peptides located in the two large extracellular loops were used to generate antibodies for Western blotting and immunogold localization. To confirm that this antibody is specific for protein WP_009059494, the recombinant protein PelB-MFUM_2087 was used and showed a positive reaction on an immunoblot (data not shown). Then, cell fractions from strain SolV were used to determine the cellular location of WP_009059494 by Western blotting with antibody against synthesized peptides. Samples from the total membrane fraction showed a positive reaction, whereas samples from the soluble fraction (including cytoplasm and periplasm) did not. Sarcosyl was used for selective solubilization of the inner membrane (Filip et al. 1973), after which detergent CHAPS was used for solubilization of the outer membrane. Treatment with Sarcosyl and CHAPS released soluble proteins (including major outer membrane proteins) that showed a positive immunoblot response, whereas release of soluble proteins (including major inner membrane proteins) with Sarcosyl only did not show a positive immunoblot response. After Sarcosyl and CHAPS treatment of membranes, a relatively clear band of about 120 kDa was obtained in SDS-PAGE (Fig. 4A, Lane 1) and this band showed a positive immunoblot reaction using the peptides antibodies. In order to further confirm this band to contain WP_009059494, this band was excised for analysis by MALDI-TOF MS. The results showed the masses of peptides predicted after trypsin digestion of WP_009059494. Based on these results, the band of around 120 kDa resembles a tetrameric form of WP_009059494. Knowledge on occurrence of oligomerization of OMP is sparse but has been reviewed (Meng et al. 2009). This led to the conclusion that it may be as common and versatile as in soluble proteins.

Since this protein was highly expressed, we wanted to know where the large amount of this protein is located. Previous experiments showed that the recombinant protein was soluble in Tris–HCl buffer with SDS (0.4%). Therefore, membrane pellets were incubated in this buffer and boiled for 30 min, followed by stirring at room temperature (RT) overnight. Such conditions were expected to dissociate the outer membrane and the outer membrane proteins. As expected, a similar band of approximately 120 kDa was obtained, which was confirmed to contain protein WP_009059494 by Western blot (Fig. 4B, Lane 2). In addition, a strong immunostained band of approximately 30 kDa was observed, however this band was not readily detectable by staining with Coomassie Brilliant Blue (Fig. 4B, Lane 2). In addition to the whole membrane, pellets including cell debris and unbroken cells, were treated in the same way and analysed by SDS-PAGE and Western blotting. A positive immunoblot reaction with the antiserum was observed in the solubilized samples (Fig. 4B, Lanes 3 and 4), indicating that protein WP_009059494 was also present in the cell debris and unbroken cell fraction, suggesting that this protein may be present on the cell surface. Overall, it seems that protein WP_009059494 was present in the OM and cell surface, and it has a strong association with the OM, as SDS and heating only released a small amount of WP_009059494 from the OM, detected by immunoblotting but not Coomassie Brilliant Blue staining.

### Immunogold localization of Protein WP_009059494 in strain SolV

**Fig. 5:**
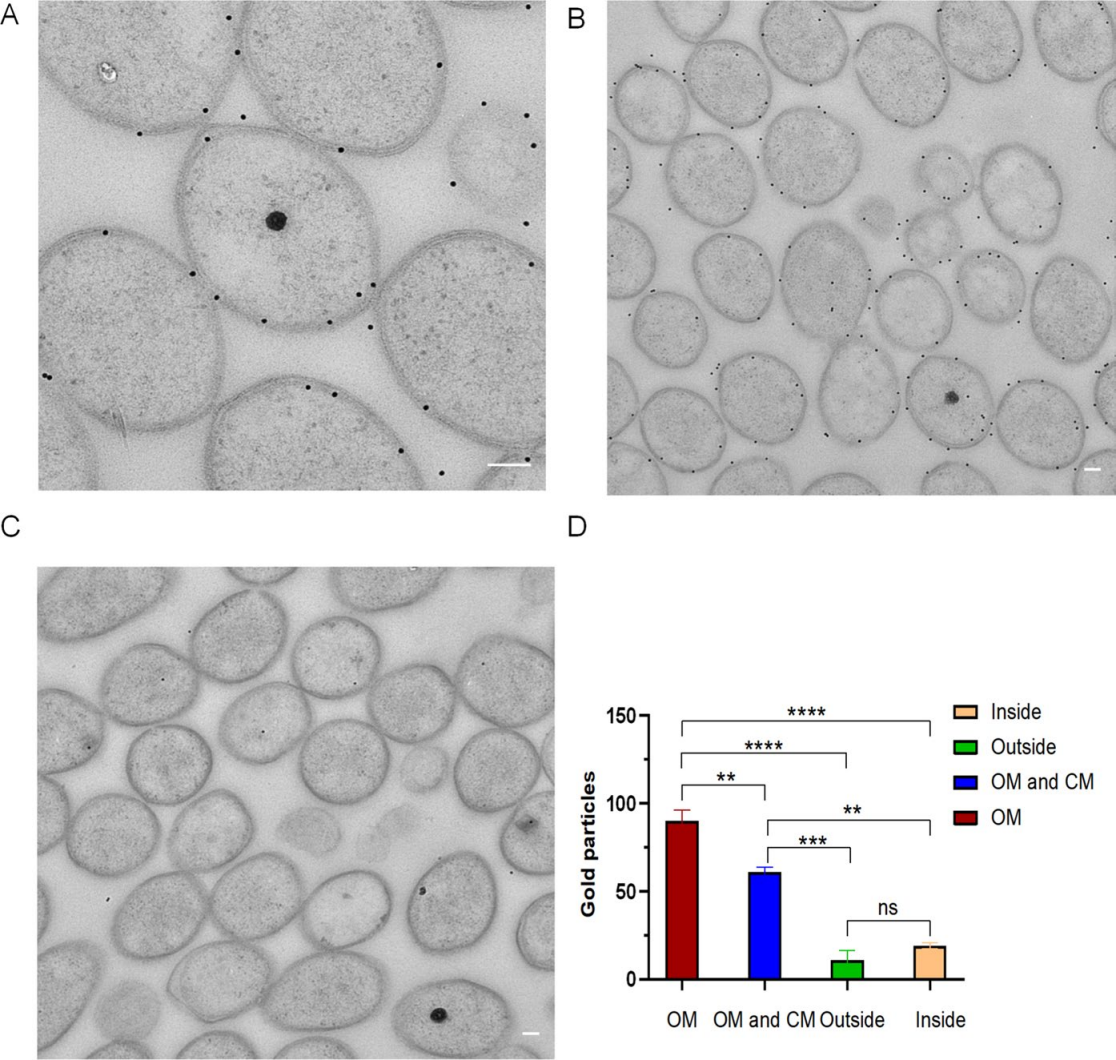
Immunogold localization of protein WP_009059494 in strain SolV. **A** and **B** Immunogold localization using the antiserum against synthetic peptides1/2 from WP_009059494 localizes the putative OMP to the outermost membrane in Lowicryl embedded cells of strain SolV. Negative control incubated with pre-immune serum instead of antiserum **C** shows only a very limited amount of background labeling. Scale bars 100 nm. **D** Statistical analysis using Graphpad Prism 8. Labeling on outer membrane (OM) vs both OM and cytoplasmic membrane (CM), *p*-value is 0.0017 (**), meaning very statistically significant; ‘OM’ vs inside the cell (‘Inside), *p*-value is 0.00004 (****), meaning extremely statistically significant; ‘OM’ vs outside the cell (Outside), *p*-value is 0.00007 (****), meaning extremely statistically significant; no statistically significant difference (ns) between the categories ‘inside’ and ‘outside’

Immunogold localization was performed on sections of high pressure frozen, freeze-substituted strain SolV cells embedded in Lowicryl HM20 resin (Fig. 5ABC). A total of 540 gold labels were quantified in 113 cells (Supplementary Fig. S1), considering that the length of an antibody-protein-A gold complex is approximately 25 nm (measured till the border of the gold particle). In this way, all labels were classified in one of four categories: “outer membrane (OM)”, “outer membrane and cytoplasmic membrane (OM and CM)”, “cytoplasm (inside)”, or “outside” based on their distance from the respective membrane. The amount of labelling in the negative control incubated with pre-immune serum was negligible (Fig. 5C). Of the 540 gold labels from 113 cells, 50% of the labels targeted an epitope of the OM. In addition, 34% of the labels were targeted to the OM and CM category. We also noted that 6% and 10% of the labels targeted a location out- or inside of the cells, respectively. These results suggest protein WP_009059494 to be predominantly present in the OM. Statistical analysis (Fig. 5D) shows that the difference in the number of labels between the categories "OM" and "OM and CM" is statistically very significant, in addition to the fact that due to the very small distance between OM and CM, many of the labels in ‘OM and CM’ are more likely linked to the OM, further suggesting that the protein is predominantly present in the OM. More labels were present in the ‘OM’ than ‘inside’ and ‘outside’, with very high statistical significance (P-values < 0.0007). No statistically significant difference could be found between the categories ‘inside’ and ‘outside’.

**Fig. 6:**
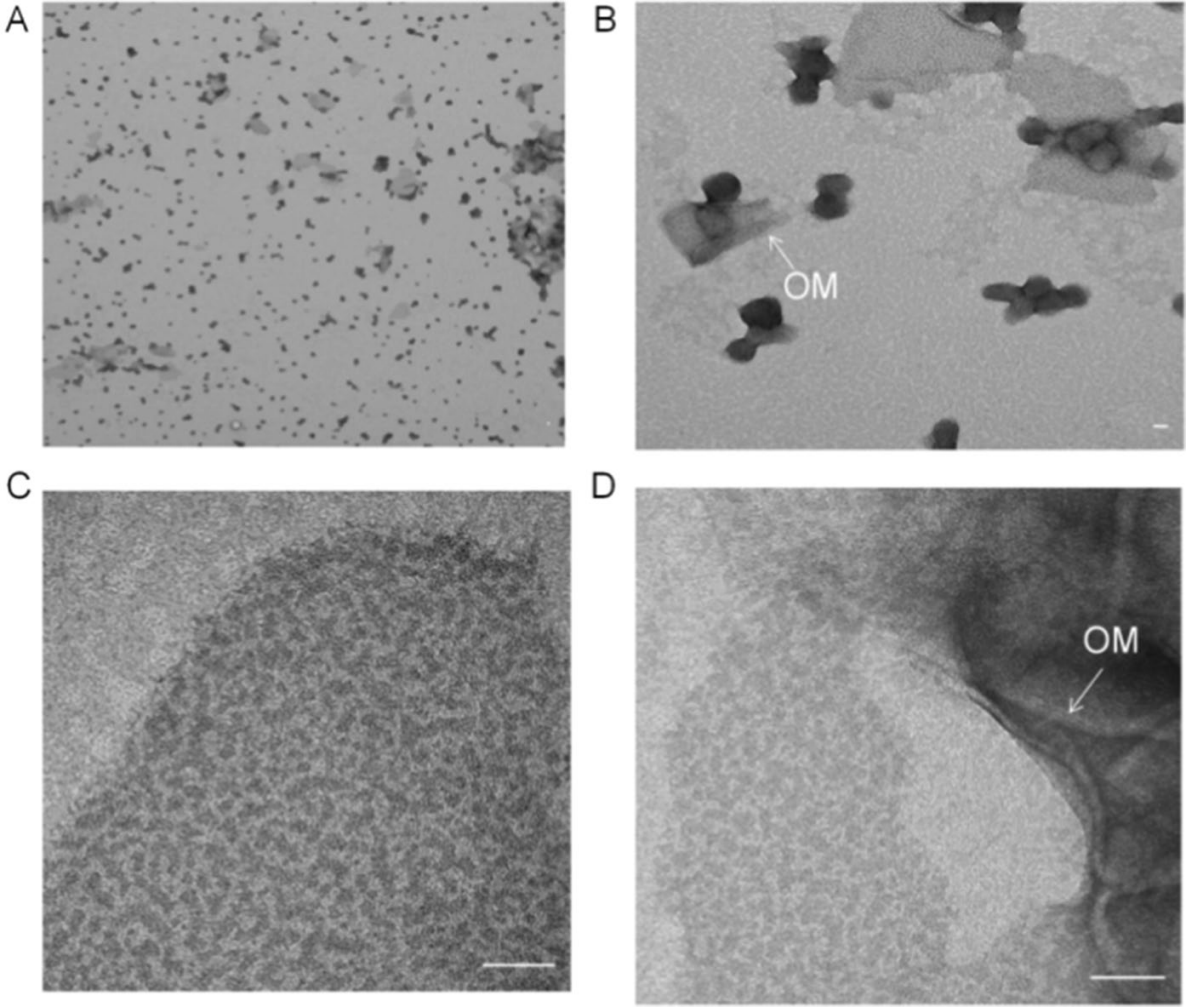
Negative staining with TEM for protein WP_009059494. Sample from Lane 2 (Fig. 4B) was used to perform negative staining with TEM, piece of outer membrane was observed. Panels show increased magnification: **A** 3000 x; **B** 20,000 x; **C** and **D** 100,000 x. Scale bars are 50 nm. This result indicates that protein WP_009059494 may form particles that do not have a regular pattern as checked by Fourier transform analysis. *OM* Outer membrane, pointing to parts were fragments of the OM are visible

As mentioned before, the protein sample visualized in Lane 2 of Fig. 4B showed a positive immunoblot reaction also at 120 kDa using the peptide antiserum. To find out if WP_009059494 can oligomerize and may be a putative S-layer protein forming a regular structure, a negative staining of this fraction by TEM analysis was performed. Figure 6 shows the sample from Lane 2 (Fig. 4B) to form protein particles that did not have a regular pattern, which is consistent with the very poor solubility, even boiling SDS could not completely dissolve it.

### The effect of overexpression of WP_009059494 on the membrane of* E. coli*

The probe NPN is a hydrophobic, non-polar compound that fluoresces strongly in a phospholipid environment, but only weakly in an aqueous environment (Helander and Mattila-Sandholm 2000). The OM is unique among biological membranes because it is able to exclude external hydrophobic molecules such as NPN. Therefore, the NPN uptake assay can be used to detect the permeability of the OM. When comparing the fluorescence values of *E. coli* expressing protein WP_009059494 with those of control *E. coli* containing the empty plasmid, the ratio was around 1.9 (Supplementary Fig. S2 AB), indicating that expression of protein WP_009059494 increased the permeability of the OM, leading to the entry of more of the hydrophobic molecule NPN into the cell.

The diSC_3_(5) release assay is based on the accumulation of this membrane potential-sensitive fluorescent dye in the IM in a proton motive force (PMF)-dependent manner, whereby the fluorescence is quenched by the dye. However, when the PMF is compromised, diSC3(5) is released from the IM, leading to an increase in fluorescence (Konovalova et al. 2016). When comparing the fluorescence values of *E. coli* expressing protein WP_009059494 with those of control *E. coli* containing the empty plasmid, the ratio was about 1.2 (Supplementary Fig. S2 CD), indicating that expression of protein WP_009059494 cause only slightly IM depolarization.

EDTA destabilizes lipopolysaccharide (LPS) interactions by chelating divalent cations, and SDS is an ionic detergent that penetrates the cell membrane and dissolves lipids and proteins therein, leading to cell lysis (Sheinfeld et al. 2017). To assess the effect of expression of WP_009059494 on the cell wall of *E. coli*, we tested the susceptibility of cells to EDTA and SDS. Expression of WP_009059494 with the PelB signal peptide made cells less sensitive to EDTA and SDS compared to the control (Supplementary Fig. S2 E). We also noted that cells expressing WP_009059494 with its original signal peptide were more sensitive to EDTA and SDS. This may be due to the fact that the original signal peptide was not resulting in translocation of WP_009059494 to the periplasm. Inclusion bodies are formed inside of the cell, resulting in a survival pressure on the cell. Taken together, the expression of WP_009059494 protein with PelB signal increased the permeability of the OM of *E. coli* and made the cells less sensitive to EDTA and SDS, thus being able to protect the cells to some extent.

## Discussion

In this study, we identified a major outer membrane protein WP_009059494 from *M. fumariolicum* SolV. This protein is expressed at high levels (RPKM, 26,731 ± 5431) in cells grown under various conditions. Expression is more than 135-fold higher compared to outer membrane protein A (OmpA, 200 RPKM). OmpA in *E. coli* is an abundant outer membrane protein, present at ~ 100,000 copies per cell (Koebnik 2002). The high expression of OmpA in *E. coli* supports several structural and physiological functions such as maintenance of cell shape and stability, involvement in biofilm formation, as an adhesion/invasion and evasion factor, as a colicin and bacteriophage receptor and as a mediator of F-dependent binding (Reusch 2012). The high expression of OM protein WP_009059494 in strain SolV indicates that it may play an important role in cell growth and survival.

*E. coli* is one of the most widely used hosts for the production of recombinant proteins. To secrete recombinant proteins into the periplasmic space or culture medium, several signal peptides are commonly used to produce extracellular recombinant proteins in *E. coli*, such as those from PelB, OmpA, PhoA, MalE, LamB and OmpC (Choi and Lee 2004; Zhang et al. 2018). In this study, we used the signal peptide from PelB. However, most of the recombinant protein WP_009059494 was present in inclusion bodies (Fig. 3A). This may be due to the nature of the amino acid composition of this protein, considering it has 41% hydrophobic amino acids. Denaturing and chaotropic agents like urea and guanidine hydrochloride have been often used to solubilize inclusion bodies (Palmer and Wingfield 2004), but they did not work for this recombinant protein. Ionic detergents such as SDS, are very effective in solubilizing membrane proteins (Seddon et al. 2004) and are also suitable for this recombinant protein, however, the use of SDS can also lead to denaturation to some extent. Removal of SDS can be achieved by transferring the protein to a denaturing detergent or lipid environment (Seddon et al. 2004). A successful example of this is active outer membrane protein PagP, which was recovered from the SDS-denatured state by adding amphipathic cosolvents such as 2-methyl-2,4-pentanediol (MPD) (Michaux et al. 2008). In this study, we have successfully purified the solubilized recombinant protein WP_009059494 using Tris–HCl buffer with SDS. Future experiments with lipid bilayers may provide information for determining the function of this protein. SDS-PAGE analysis showed its molecular weight to be around 35 kDa, slightly larger than the expected molecular mass (around 31–32 kDa). In addition, a band at around 20 kDa was also identified as WP_009059494 by MALDI-TOF MS. This result may be due to a change in its conformation, such as loss of β-barrel structure or incomplete folding or partial degradation. Antibodies were generated against purified recombinant protein PelB-MFUM_2087 and were shown to bind to this recombinant protein. Only the surface protein fraction from strain SolV showed a minor reaction with this antibody (Fig. 3C, Lane 7), and the antibody did not work in immunogold localization.

Based on prediction of PRED-TMBB, the WP_009059494 is a ten-stranded β-barrel integral OMP with two large extracellular loops toward the outside of the cell (Fig. 1A). Extracellular loops are common in some OMPs, such as “opacity-associated” (Opa) proteins from Gram-negative bacterial pathogens like *Neisseria gonorrheae* (Fox et al. 2014). These extracellular loops interact with host receptors to induce phagocytosis. The two large extracellular loops of WP_009059494 are specific for this protein and two short peptides in these loops were used to raise antibodies for immunoblotting and localization. Antibodies against the peptides were more effective than antibodies against the recombinant protein. Positive signals were obtained with outer membrane fraction and cell debris (Fig. 4AB), indicating that the protein was present in the OM. A band of approximately 120 kDa in SDS-PAGE was identified by Western blot and MALDI-TOF MS as containing the WP_009059494 protein (Fig. 4B, Lane 2), indicating a tetrameric configuration. In addition, a strong positive reaction was observed for a band of approximately 30 kDa, however this band was not easily detected by Coomassie Brilliant Blue staining (Fig. 4B, Lane 2), indicating that even SDS and heating were only able to release a small amount of protein WP_009059494 from the OM. Immunogold labelling with TEM provided strong evidence that protein WP_009059494 is predominantly present in the OM, facing the external environment, which is consistent with the results of immunoblotting.

Based on the PRED-TMBB and AlphaFold2 prediction, WP_009059494 forms a porin with ten antiparallel β-barrels with a small amphipathic N-terminal α-helix on the periplasmic side of the membrane. This β-barrel has a hydrophobic outer surface dominated by aromatic amino acids, and a hydrophilic interior (Fig. 1B). This structure allows the protein to be inserted into the OM and remain stable. Porins in the OM are mainly involved in the passive transport of hydrophilic molecules and are impermeable to large and charged molecules (Achouak et al. 2001). To determine whether the protein is a pore protein and what type of compounds can pass, future electrophysiological studies can provide information on the size, conductivity, selectivity, and other channel properties of protein WP_009059494. In addition, S-layers consisting of the most abundant porin is one of the most commonly observed components of the prokaryotic cell membrane (Gerbino et al. 2015; Sára and Sleytr 2000; Sleytr et al. 2014). For example, the pore protein P100 from *Thermus thermophilus* forms the S-layer, which consists entirely of this pore protein (Castón et al. 1993). In general, S-layer proteins contain 40–60% hydrophobic amino acids with few or no sulphur-containing amino acids (Sára and Sleytr 2000; Sleytr et al. 2014). The S-layer is resistant to degradative attack and can only be extracted under harsh conditions such as high temperatures in combination with strong detergents such as SDS (Gerbino et al. 2015; Sleytr et al. 2014). Protein WP_009059494 also shows these characteristics, which led us to assume that it could form S-layers on the cell surface. However, previously published freeze etching images of strain SolV (van Teeseling et al. 2014b) did not show S-layer structures. In addition, TEM after negative staining of cells from strain SolV and protein WP_009059494 did not detect a regular lattice (Fig. 6). Instead, protein WP_009059494 formed particles, suggesting that the protein is most likely an OMP. However, it still may be functionally similar forming a protective coat or molecular sieve. Unfortunately, as there is no genetic system for *M. fumariolicum* SolV, it was not possible to make a knockout mutant for this protein to assess its function. Besides surface-exposed loops, N-terminal α helices are functionally important in the OMP PagP, this N-terminal part stabilizes the folded protein in the lipid bilayer (Huysmans et al. 2007, 2010). Several other OMPs have functional domains extending in the periplasm, serving as an interaction site for proteins or peptidoglycan components like FhuA and OmpA, or being involved in the regulation of dimerization like OMPL As or stabilizing the oligomeric state by the formation of a coiled coil like in the sucrose porin ScrY (Dekker 2000; Forst et al. 1998).

The localization of WP_009059494 to the outermost membrane raised the question how this protein would be transported to the OM. After protein WP_009059494 is synthesized, the SecYEG translocon is expected to transport the unfolded proteins over the IM. When unfolded OMPs enter the periplasm, they tend to aggregate. To prevent their misfolding, periplasmic chaperones (SurA, Skp and DegP) interact with unfolded OMP and escort them through the periplasmic space to the β-barrel assembly machinery (BAM complex) that folds and inserts the nascent OMPs into the OM (Rollauer et al. 2015). However, it is unknown how the BAM complex performs its function in *Methylacidiphilum. sp*. Recently, we proposed a lipoprotein transport system (Liu et al. 2023). Several genes lacking in the OMPs and lipoprotein transporter system may indicate strain SolV use a different and relatively simple system to perform this transport.

In conclusion, the highly expressed gene *MFUM_2087* encodes an outer membrane protein WP_009059494 with an OMP-specific ß-barrel structure. The protein is highly resistant to extraction but could be purified from native *M. fumariolicum* SolV biomass and from recombinant *E. coli*. Immunogold localization confirmed the presence of this protein in the outer membrane of *M. fumariolicum* SolV. Considering the protein to have a tight association with the OM and the specific ecological niche of *M. fumariolicum* SolV living in a geothermal environment with low pH and high temperatures, it is likely that this major OMP acts as physical barrier in these extreme environmental conditions.

## Supplementary material

The Supplementary Material for this article can be found online.

### Supplementary Information

Below is the link to the electronic supplementary material.

Supplementary file1 (PDF 423 KB)

Supplementary file2 (XLSX 21 KB)

## Data Availability

On request raw data supporting the conclusions of this article will be made available by the authors, without any reservation.
