# Supplementary material for: Identification and characterisation of a major outer membrane protein from Methylacidiphilum fumariolicum SolV
Source: Antonie Van Leeuwenhoek. 2023 Sep 22;116(11):1227–45. doi: 10.1007/s10482-023-01879-0 (PMC10542722; doi:10.1007/s10482-023-01879-0)
Supplement: Supplementary file 1 — Supplementary file1 (PDF 423 KB) [file 10482_2023_1879_MOESM1_ESM.pdf]

Antonie van Leeuwenhoek

Supplementary Material

Identification and characterisation of a major outer membrane protein from *Methylacidiphilum fumariolicum* SolV

Changqing Liu[1], Rob Mesman[1], Arjan Pol[1], Federica Angius[1] & Huub J.M. Op den Camp[1]*

[1]Department of Microbiology, Radboud Institute for Biological and Environmental Sciences, Faculty of Science, Radboud University, Nijmegen, the Netherlands

*** Correspondence:** h.opdencamp@science.ru.nl

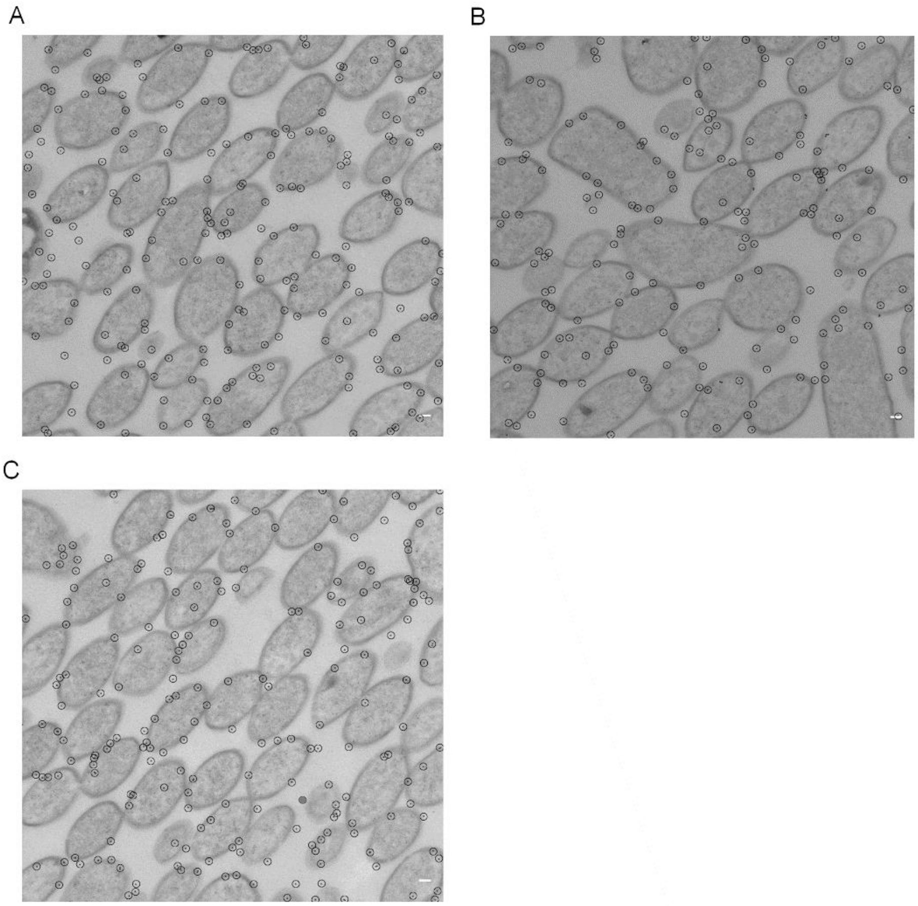

Supplementary Fig. S1. Immunogold localization analysis in strain SolV.

(A-C) Immunogold localization of the antiserum against peptide1/2 from WP_009059494 localizes the putative OMP to the outermost membrane of strain SolV cells embedded in Lowicryl HM20. All gold labels are surrounded by a circle that spans 25 nm from the edge of the gold particle, to show the possible epitopes that the particle labels, scale bars 100 nm.

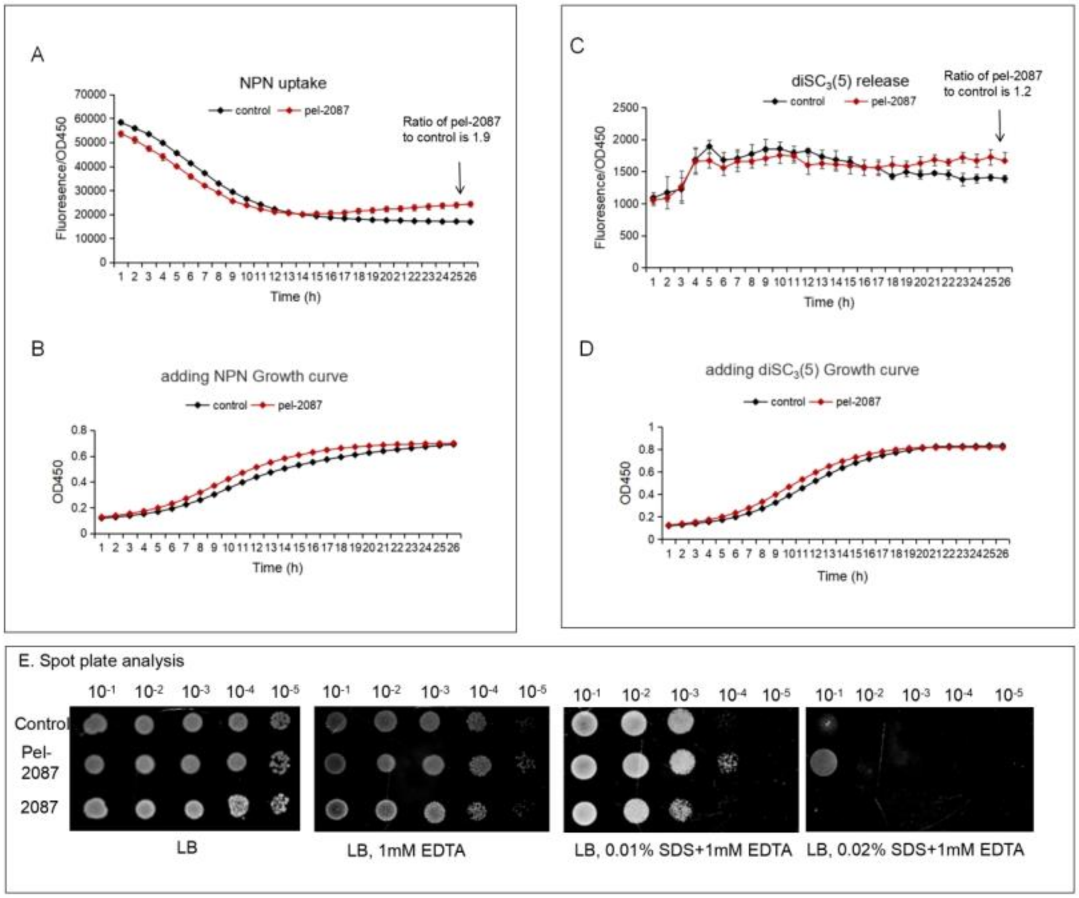

Supplementary Fig. S2. Overexpression of WP_009059494 (MFUM_2087) effect on the growth of *E. coli* cells.

(AB) Cellular fluorescence intensity varies after adding NPN and cell growth curve with addition of NPN. Control represents *E. coli* containing an empty plasmid. It should be noted that the cellular fluorescence intensity of *E. coli* cells containing pET-28a-pel-*MFUM_2087*, increased 90% comparing to the control *E. coli* cells containing an empty plasmid, implying that the expression of WP_009059494 had some effect on the outer membrane (OM). (CD) Cellular fluorescence intensity varies after adding diSC$_3$(5) and cell growth curve with addition of diSC$_3$(5). Control represents *E. coli* containing an empty plasmid. It should be noted that the cellular fluorescence intensity of *E. coli* cells containing pET-28a-*pel-MFUM_2087*, increased 20% comparing to the control *E. coli* cells containing an empty plasmid, implying that the expression of WP_009059494 had no effect on the outer membrane (OM). (E) Overnight cultures grown in LB supplemented with different cells (containing different plasmids), normalised to an OD_{600} of 1.00 and 10-fold serially diluted before inoculating LB agar plates supplemented with SDS (0.01% or 0.02%) and EDTA (1 mM).
